# Machine learning algorithms identifying the risk of new-onset ACS in patients with type 2 diabetes mellitus: A retrospective cohort study

**DOI:** 10.3389/fpubh.2022.947204

**Published:** 2022-09-06

**Authors:** Zuoquan Zhong, Shiming Sun, Jingfan Weng, Hanlin Zhang, Hui Lin, Jing Sun, Miaohong Pan, Hangyuan Guo, Jufang Chi

**Affiliations:** ^1^Department of Cardiology, Shaoxing People's Hospital, Shaoxing Hospital of Zhejiang University, Shaoxing, China; ^2^The First Clinical Medical College, Wenzhou Medical University, Wenzhou, China; ^3^Department of Cardiology, Zhejiang University School of Medicine, Hangzhou, China; ^4^College of Medicine, Shaoxing University, Shaoxing, China

**Keywords:** type 2 diabetes mellitus, acute coronary syndrome, machine learning, random forest, nomogram

## Abstract

**Background:**

In recent years, the prevalence of type 2 diabetes mellitus (T2DM) has increased annually. The major complication of T2DM is cardiovascular disease (CVD). CVD is the main cause of death in T2DM patients, particularly those with comorbid acute coronary syndrome (ACS). Although risk prediction models using multivariate logistic regression are available to assess the probability of new-onset ACS development in T2DM patients, none have been established using machine learning (ML).

**Methods:**

Between January 2019 and January 2020, we enrolled 521 T2DM patients with new-onset ACS or no ACS from our institution's medical information recording system and divided them into a training dataset and a testing dataset. Seven ML algorithms were used to establish models to assess the probability of ACS coupled with 5-cross validation.

**Results:**

We established a nomogram to assess the probability of newly diagnosed ACS in T2DM patients with an area under the curve (AUC) of 0.80 in the testing dataset and identified some key features: family history of CVD, history of smoking and drinking, aspartate aminotransferase level, age, neutrophil count, and Killip grade, which accelerated the development of ACS in patients with T2DM. The AUC values of the seven ML models were 0.70–0.96, and random forest model had the best performance (accuracy, 0.89; AUC, 0.96; recall, 0.83; precision, 0.91; F1 score, 0.87).

**Conclusion:**

ML algorithms, especially random forest model (AUC, 0.961), had higher performance than conventional logistic regression (AUC, 0.801) for assessing new-onset ACS probability in T2DM patients with excellent clinical and diagnostic value.

## Introduction

Type 2 diabetes mellitus (T2DM) is a prevalent chronic disease with an increasing worldwide increase. In 2018, ~11% of the world's population was diagnosed with diabetes, with a large proportion of patients being undiagnosed in China (1). The complications of T2DM, not T2DM alone, have terrible consequences. Diabetes complications include microangiopathy or macroangiopathy as well as the cardiovascular and nervous systems (2). A study showed that, compared to patients without T2DM, those with T2DM suffer from high-risk cardiovascular factors a mean 14.6 years earlier (3).

A high blood glucose level as an independent cardiovascular risk factor increases the risk of acute coronary syndrome (ACS) (4–6). The main mechanisms of ACS are rupture or the invasion of coronary atherosclerotic plaques and secondary occlusive thrombosis, including acute ST-segment elevation myocardial infarction, acute non-ST segment elevation myocardial infarction, and unstable angina pectoris (7). There were 17.92 million deaths due to coronary heart disease in 2015 (8). A decline in patient productivity and improvement in rehospitalization probability due to ACS caused huge economic losses (9). High glucose levels are strongly associated with low-density lipoprotein cholesterol related to ACS, and adults with T2DM have a much higher probability of ACS than those without ACS (10). More attention should be paid to the probability of ACS in T2DM patients, and a prediction model should be established for the arm as soon as possible. Machine learning (ML) can overcome the limitations of the above problem. ML, an interdisciplinary subject based on artificial intelligence, studies how computers learn from data and continuously improve its performance (11). In recent years, many ML algorithms (12–14) have been used to establish a prediction model for diagnosing cardiovascular disease (CVD) and determining patient prognosis. A single algorithm often has its own advantages or disadvantages and cannot satisfy all of the data. Conversely, using different ML method algorithms can greatly improve the prediction ability and identify the best prediction model. Therefore, our key characteristics of the ML method include traditional logistic regression and other ML method algorithms.

## Materials and methods

### Study population

This observational retrospective cohort study collected data from 521 patients diagnosed with T2DM at Shaoxing People's Hospital from January 2019 to January 2020. The academic ethics committee of Shaoxing People's Hospital approved the study protocols, and all participants completed informed consent. According to the International Classification of Diseases (ICD)-10 (120.0, 121, 122), the diagnostic criteria of ACS included ST-segment elevation myocardial infarction, non-ST segment elevation myocardial infarction, or unstable angina pectoris. The diagnosis of T2DM was as follows: (1) a random venous plasma glucose concentration ≥ 11.1 mmol/L; (2) fasting blood glucose concentration ≥ 7.0 mmol/L (whole blood ≥ 6.1 mmol/L) or 2-h plasma glucose concentration ≥ 11.1 mmol/L after an oral glucose tolerance test; (3) glycosylated hemoglobin A1c (HbA1c) level ≥ 6.5%. Moreover, we excluded other types of diabetes, such as type 1, gestational, monogenic, and drug- or chemically induced. Among the 521 enrolled patients from the Chest Pain Center of Shaoxing People's Hospital, 222 were diagnosed with T2DM and new-onset ACS, while the other 299 were diagnosed with T2DM but not ACS. Patients with T2DM were excluded if they had: (1) a history of myocardial infarction and stent implantation; (2) a history of cancer or tumor resection; (3) rheumatic or immunological diseases; (4) severe liver failure or disseminated intravascular coagulation with concomitant severe infection and renal failure; (5) a history of stroke.

### Data collection

A total of 39 clinical and demographic characteristics were collected by trained clinicians from the medical information recording system of Shaoxing People's Hospital. Demographic features included sex; age; history of smoking, drinking, hypertension, or hyperlipidemia; and family history of CVD (myocardial infarction, stroke, hypertension, heart failure, peripheral artery disease, etc.). Clinical data comprised respiratory rate; heart rate; systolic blood pressure; diastolic blood pressure; Killip grade; and serum biomarkers including aspartate aminotransferase (AST), lactate dehydrogenase, total bilirubin, total protein, albumin, globulin, albumin/globulin ratio, urea, creatinine, uric acid, total cholesterol, triglyceride (TG), high-density lipoprotein, low-density lipoprotein cholesterol, apolipoprotein A1, apolipoprotein B, apolipoprotein B/apolipoprotein A1, fasting blood glucose (FBG), α-hydroxybutyrate dehydrogenase, creatine kinase MB, homocysteine, C-reactive protein, neutrophil count, lymphocyte count, neutrophil-lymphocyte ratio, HbA1c, and triglyceride-glucose (TyG) index: ln [fasting TG (mg/dL) × FBG (mg/dL)/2]. Complete clinical and demographic characteristics were available for all patients. All characteristics were collected within 24 h of the patients' hospitalization. Patients for whom complete data were missing were excluded to ensure high data integrity.

### Statistical analysis

Normally distributed data are presented as mean ± standard deviation, and the differences between the two groups were compared using an independent sample *t*-test. Classified data are described as counts (percentages), and the Pearson chi-square test (Pearson χ^2^ test) was used to compare the classification variables. Correlations between the 39 features were examined using the Pearson correlation test or Spearman's rank correlation test.

The initial dataset was randomly divided into a training dataset and a testing dataset at a ratio of 70:30. The training dataset was used to create and validate the models, the robustness of which were verified by the testing dataset. Significant features with values of *P* ≤ 0.05 were selected from the training dataset using the least absolute shrinkage and selection operator (LASSO) approach. The features chosen by the LASSO approach and other clinical characteristics were used to perform the multivariate logistic regression and establish the prediction models in the training dataset. The selected features were incorporated into the nomogram to predict the probability of new-onset ACS in patients with T2DM. The area under the curve (AUC) of the receiver operator characteristic (ROC) curve analysis was used to assess the discriminatory capacity of the nomogram. In addition, a calibration curve was constructed in the training group to predict the similarity between the prediction probability and the actual observed probability. Moreover, decision curve analysis was used to evaluate the clinical usefulness of the nomogram by quantifying the net benefits at different threshold probabilities.

Six other common ML algorithms (K-nearest neighbor [KNN], support vector machine [SVM], decision tree, random forest, extreme gradient boosting, and artificial neural networks [ANN]) developed prediction models for the probability of new-onset ACS. All models were coupled with 5-cross validation. The KNN model was classified by measuring the distance between different feature values, used the training data to divide the feature vector space, and considered the division result the final algorithm model (15). The SVM is a generalized linear classifier that performs binary data classification in a supervised learning method, treats each predictor as a dimension in a high-dimensional space, and tries to identify the best hyperplane to classify the sample (16). The decision tree is a tree structure in which each internal node represents a judgment on an attribute, each branch represents the output of a judgment result, and each leaf node represents a classification result (17). The random forest is a classifier containing multiple decision trees. The algorithm classifies the input vectors. Each tree is classified, and the input vector should be “voted.” The forest is the tree that chooses the most votes (18). Artificial neural networks imitate the behavioral characteristics of animal neural networks and adjust the connection between internal nodes to process information on the system's complexity (19).

**Figure 1:**
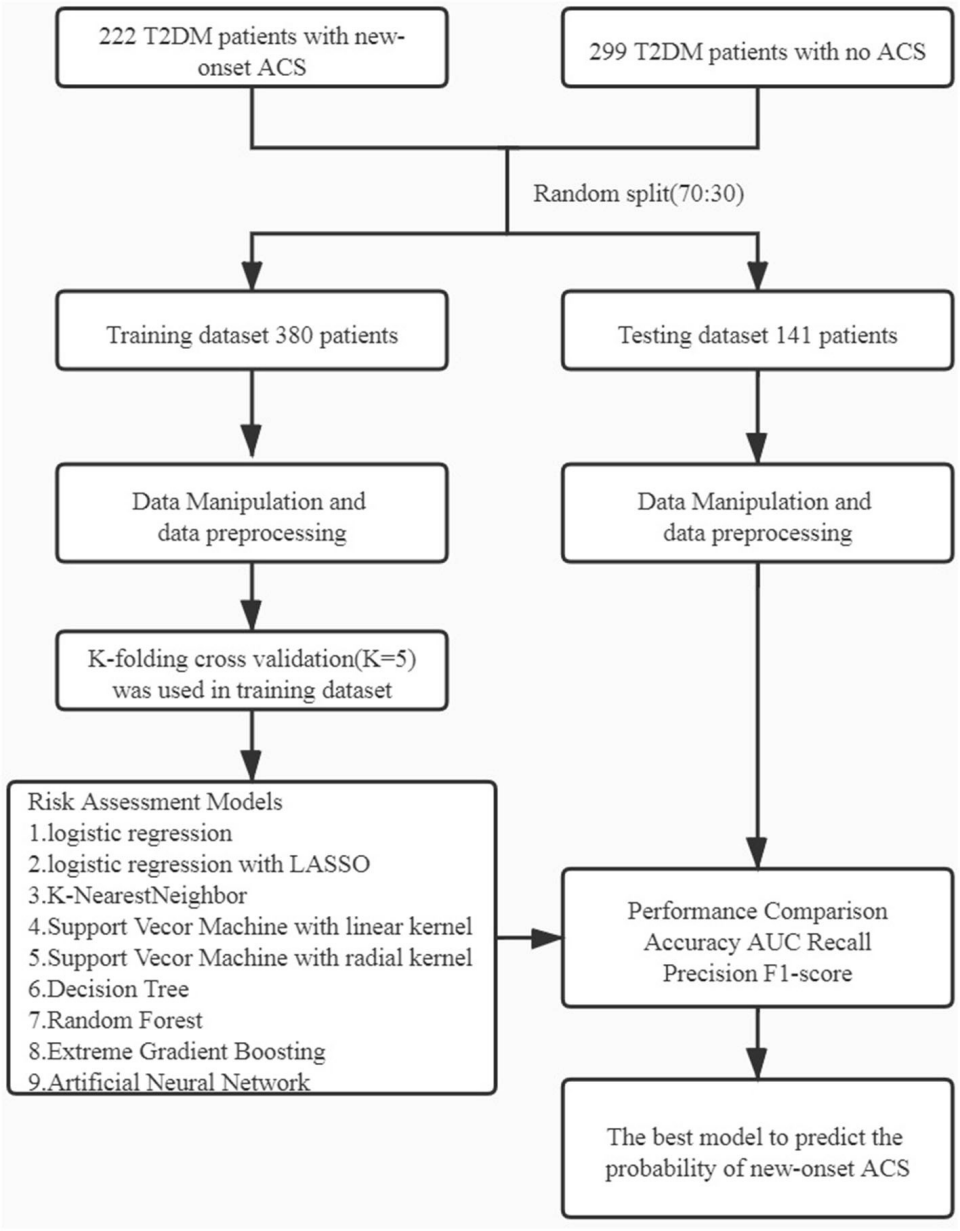
Workflow diagram: The initial dataset was randomly split into training dataset and testing dataset in the ratio of 70:30. Different machine learning algorithms were using k-folding cross validation (k = 5). ACS, acute coronary syndrome.

All performance parameters (accuracy, AUC, recall [sensitivity], precision, and F1 score) were recorded for the training and testing datasets (Figure 1). All data analyses and ML models were performed using R version 4.1.0 (The R Foundation for Statistical Computing, Vienna, Austria). All of the statistical tests were two-tailed, and values of *P* < 0.05 were considered statistically significant.

## Result

### Baseline characteristics

A total of 521 patients with T2DM were enrolled in this study. Of them, 222 were newly diagnosed with ACS, while the other 299 did not have ACS. The baseline characteristics of the training and testing datasets are presented in Table 1, and the feature correlation heatmap is shown in Supplementary Figure 1. There were no statistically significant intergroup differences except for a history of drinking (*P* = 0.031), history of hyperlipidemia (*P* = 0.019), TG level (*P* = 0.003), and TyG index: ln [fasting TG (mg/dL) × FBG (mg/dL)/2] (*P* = 0.014).

**Table 1:** Patients characteristics.

**Baseline characteristics**	**Training dataset (*n* = 380)**	**Testing dataset (*n* = 141)**	***P* value**
Sex			
1 = Male, *n* (%)	237 (62)	80 (57)	0.242
2 = Female, *n* (%)	143 (38)	61 (43)	
Age, years	64.3 ± 12.3	65.1 ± 12.0	0.54
Smoking, *n* (%)			
1 = YES	135 (36)	49 (35)	0.869
0 = NO	245 (64)	92 (65)	
Drinking, *n* (%)			
1 = YES	135 (36)	36 (26)	0.031
0 = NO	245 (64)	105 (74)	
Breath, times/min	18.8 ± 1.8	19.2 ± 5.7	0.231
Heartrate, beats/min	82.4 ± 14.0	80.3 ± 14.2	0.127
SBP, mm/hg	138.6 ± 21.1	138.4 ± 18.2	0.932
DBP, mm/hg	81.0 ± 12.1	79.5 ± 10.7	0.199
Killip, *n* (%)			
1	340 (89)	127 (90)	0.898
2	28 (7)	9 (7)	
3	9 (3)	3 (2)	
4	3 (1)	2 (1)	
Hypertension, *n* (%)			
1 = YES	228 (60)	76 (54)	0.21
0 = NO	152 (40)	65 (46)	
Hyperlipidemia, *n* (%)			
1 = YES	153 (40)	41 (29)	0.019
0 = NO	227 (60)	100 (71)	
Family history of CVD, *n* (%)			
1 = YES	41 (11)	9 (6)	0.129
0 = NO	339 (89)	132 (94)	
AST, U/L	49.0 ± 83.6	47.1 ± 83.2	0.818
LDH, U/L	268.7 ± 242.1	264.2 ± 239.2	0.849
TBIL, umol/L	12.6 ± 7.4	11.9 ± 5.1	0.217
Total protein, g/L	65.2 ± 6.4	11.9 ± 15.1	0.371
Albumin, g/L	38.5 ± 4.8	38.6 ± 3.8	0.921
Globulin, g/L	26.7 ± 4.5	27.1 ± 4.0	0.324
A/G	1.5 ± 1.2	1.5 ± 0.3	0.408
Urea, mmol/L	6.0 ± 3.4	5.8 ± 2.4	0.555
Creatinine, umol/L	78.2 ± 67.7	72.9 ± 32.3	0.367
Uric acid, umol/L	322.2 ± 108.5	308.1 ± 96.3	0.179
Total cholesterol, mmol/L	4.5 ± 1.3	4.5 ± 1.1	0.513
Triglyceride, mmol/L	1.9 ± 2.0	1.5 ± 0.9	0.003
HDL, mmol/L	1.1 ± 0.3	1.1 ± 0.4	0.297
LDL, mmol/L	2.7 ± 0.9	2.7 ± 0.9	0.881
Apo A1, g/L	1.1 ± 0.2	1.1 ± 0.3	0.595
Apo B, g/L	1.0 ± 0.3	1.0 ± 0.3	0.415
Apo B/Apo A1	0.9 ± 0.3	0.9 ± 0.3	0.328
Fasting blood glucose, mmol/L	9.8 ± 4.2	9.2 ± 3.0	0.108
HBDH, U/L	206.3 ± 210.9	210.3 ± 232.2	0.854
CKMB, U/L	26.2 ± 47.1	19.9 ± 26.2	0.054
Homocysteine, umol/L	11.2 ± 5.2	12.7 ± 18.0	0.128
C-reactive protein, mg/L	14.7 ± 27.2	14.1 ± 28.5	0.836
Neutrophils,*10^12^/L	4.3 ± 2.4	4.0 ± 2.1	0.319
Lymphocyte,*10^12^/L	1.7 ± 0.8	1.7 ± 0.6	0.688
Neutrophils/lymphocyte	3.3 ± 3.8	2.9 ± 2.8	0.312
HbA1c, %	8.4 ± 2.2	8.5 ± 2.0	0.609
TyR	9.3 ± 0.8	9.1 ± 0.6	0.014

Variables with normal distribution were presented as mean ± standard deviation (SD), other variables with classification were described as counts (percentages). Abbreviations: SBP, systolic blood pressure; DBP, diastolic blood pressure; CVD, cardiovascular disease; AST, aspartate aminotransferase; LDH, lactate dehydrogenase; TBIL, total bilirubin; A/G, albumin-globulin ratio; HDL, high-density lipoprotein; LDL, low-density lipoprotein; Apo A1,apolipoprotein A1; Apo B, apolipoprotein B; Apo B/Apo A1, apolipoprotein B-apolipoprotein A1 ratio; HBDH,α- hydroxybutyrate dehydrogenase; CKMB, creatine kinase MB; Neutrophils/lymphocyte, neutrophil-lymphocyte ratio; TyG, ln [fasting TG (mg/dL) × FBG (mg/dL)/2].

### Feature construction and summarization

According to LASSO logistic regression analysis, six of 39 features were potential indicators in the training dataset to predict the possibility of ACS in T2DM patients (Supplementary Figure 2). The selected features were a family history of CVD, a history of drinking, age, neutrophil count, Killip grade, and AST. Given the harmful effects of smoking on the cardiovascular system (20), a history of smoking was also enrolled as a potential indicator. To calculate the regression coefficient, odds ratios, and *P* values of the potential indicators, we made multivariate logistic regression shown that a family history of CVD (OR, 8.302; 95% CI, 3.566–19.326; *P* < 0.0001), history of smoking (OR, 1.819; 95% CI, 0.994–3.327; *P* = 0.0523), history of drinking (OR, 0.310; 95% CI, 0.163–0.592; *P* = 0.0004), age (OR, 3.261; 95% CI, 2.075–5.127; *P* < 0.0001), neutrophil count (OR, 1.488; 95% CI, 1.09–2.031; *P* = 0.0122), Killip grade (OR, 159.060; 95% CI, 9.545–2,584.400; *P* = 0.0004),40 ≤ AST < 200 (OR, 8.557; 95% CI, 3.721–19.676; *P* < 0.0001) and AST ≥ 200 (OR, 47.548; 95% CI, 4.852–466.01; *P* = 0.0009) were associated with T2DM patients with new-onset ACS (Table 2).

**Table 2:** Multivariate logistic regression analysis.

**Intercept and variable**	**Coefficient**	**Odds ratio (95%CI)**	***P*-value**
(Intercept)	−7.689		<0.0001
Family history of CVD	2.116	8.302 (3.566–19.326)	<0.0001
**Smoking**			
0 = NO	Reference		
1 = YES	0.598	1.819 (0.994–3.327)	0.0523
**Drinking**			
0 = NO	Reference		
1 = YES	−1.170	0.310 (0.163–0.592)	0.0004
Age	0.066	3.261 (2.075–5.127)	<0.0001
Neutrophils	0.175	1.488 (1.090–2.031)	0.0122
Killip	1.686	157.060 (9.545–2,584.400)	0.0004
AST <40	Reference		
AST 40-200	2.147	8.557 (3.721–19.676)	<0.0001
AST ≥ 200	3.862	47.548 (4.852–466.010)	0.0009

### Construction of nomogram

**Figure 2:**
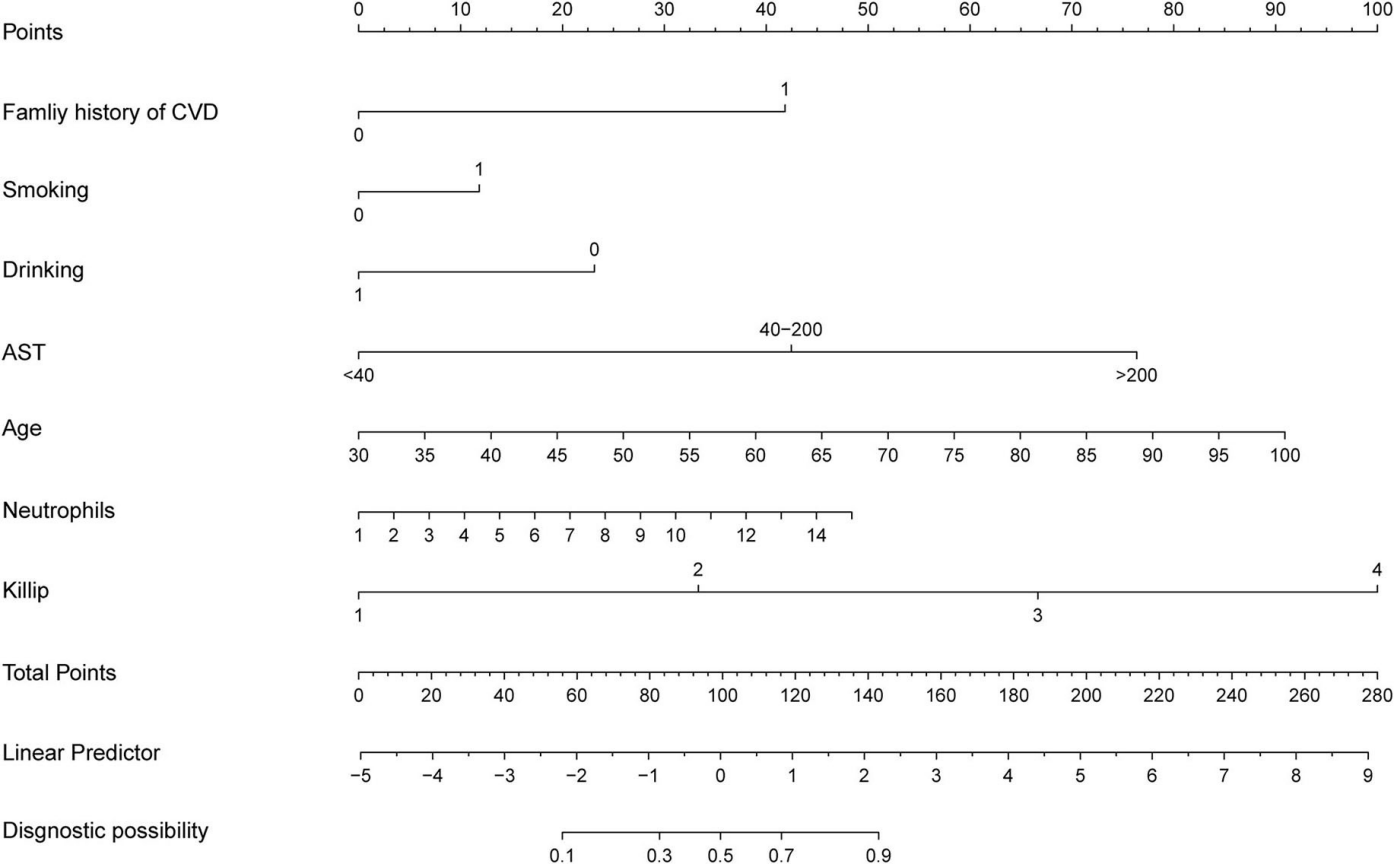
Developed newly ACS prediction nomogram in T2DM patients. ACS, acute coronary disease; CVD, cardiovascular disease; AST, aspartate aminotransferase.

As shown in Figure 2, a nomogram incorporating the above features to calculate the possibility of T2DM patients with ACS was constructed. The nomogram performed a C-index of 0.86 (95% CI, 0.82–0.90) in the training dataset vs. 0.80 (95% CI, 0.73–0.80) in the testing dataset, which could describe the model's predictive ability by considering the occurrence of the results (21). Moreover, the calibration curve showed consistency between the actual diagnosis of ACS and its predicted probability (Supplementary Figure 3).

### Assessment of nomogram

The decision curve analysis was based on the net benefit under event threshold probabilities for the nomogram to forecast the probabilities of ACS (22). Supplementary Figure 4 shows that remarkable net benefits were obtained with the nomogram in the training and testing datasets, which indicated the nomogram's clinical validity.

### Model performance of ML algorithms

**Figure 3:**
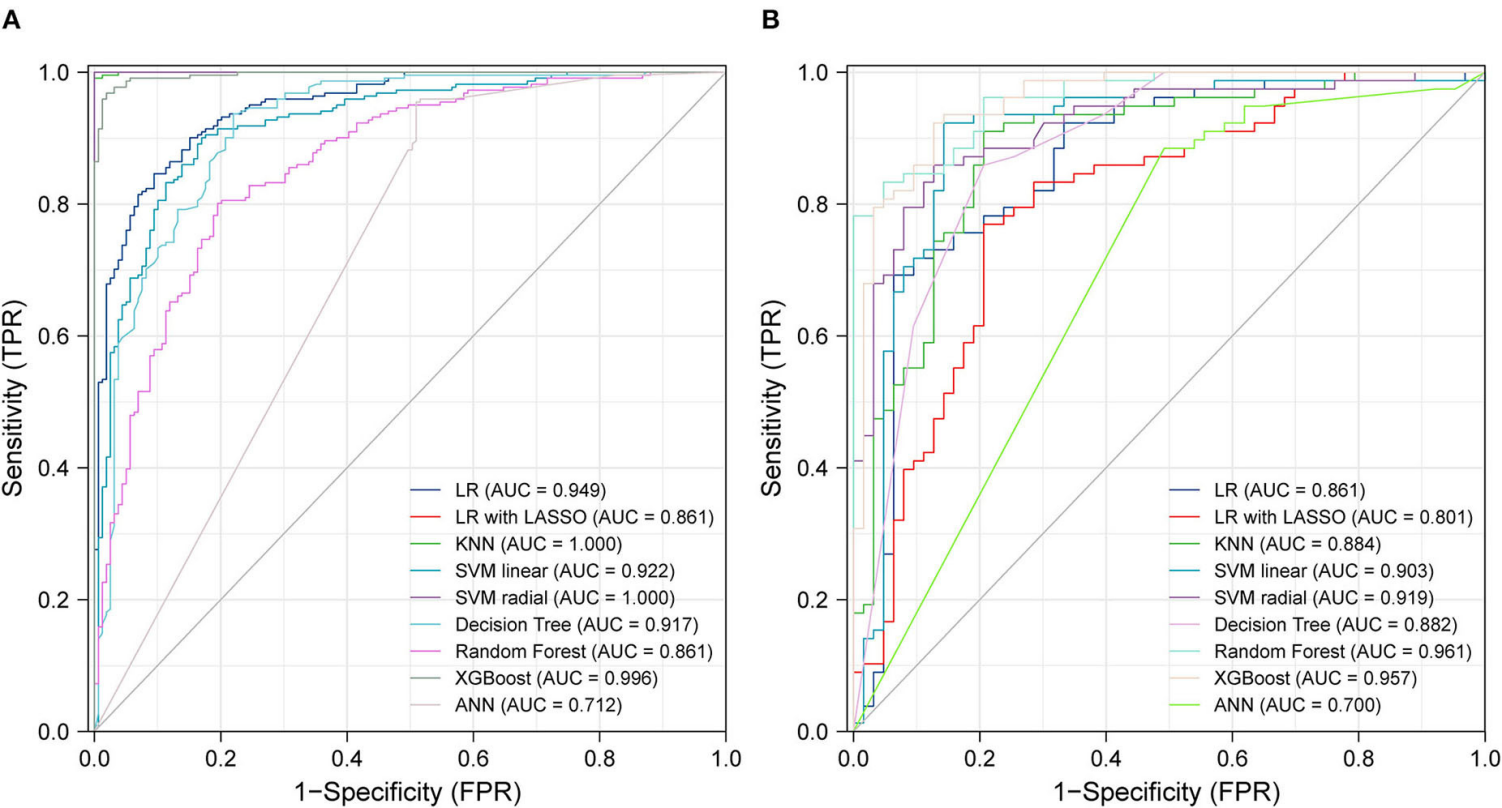
ROC curves from training test **(A)** and testing test **(B)** using different machine learning algorithms. Legend including area under receiver operator characteristic curve for each algorithm with 95% confidence intervals. LR, logistic regression; LASSO, the least absolute shrinkage and selection operator; KNN, K-nearest neighbor; SVM, support vector machine; XGBoost, extreme gradient boosting; ANN, artificial neural networks.

Figure 3A shows the ROC curves for various ML methods in the training dataset. The highest AUC (1.00) under the ROC curve was achieved using the KNN model and the SVM with radial kernel model, and the 95% CI values were 0.99–1.00 and 1.00–1.00, respectively. The logistic regression model, logistic regression with LASSO model, SVM with linear kernel model, decision tree model, random forest model, extreme gradient boost model, and artificial neural network model also performed well with AUC values of 0.7–1, representing excellent diagnostic ability. Extreme gradient boosting was the most consistent method, with an AUC of 1.00 (95% CI, 0.99–1.00) in the training dataset and 0.96 (95% CI, 0.93–0.99) in the testing dataset (Figure 3B). The prediction ability of the KNN and SVM models with the radial kernel model, which performed best in the training dataset, decreased in the testing dataset with an AUC of 0.96 (95% CI, 0.93–0.99) and 0.92 (95% CI, 0.87–0.97), respectively. Other methods such as logistic regression, SVM with linear kernel model, decision tree, and artificial neural network were well displayed in the testing dataset.

Table 3 presents the accuracy, AUC, recall, precision, and F1 score of the different ML methods in both training and testing datasets. SVM with a radial kernel model (accuracy, 0.99; AUC, 1.00; recall, 0.98; precision, 1.00; F1 score, 0.99) demonstrated the highest performance in the training dataset. In the testing dataset, the highest performing model was the random forest (accuracy, 0.89; AUC, 0.96; recall, 0.83; precision, 0.91; F1 score, 0.87).

**Table 3:** The contrast of different machine learning models performance.

**Model**	**Training dataset**					**Testing dataset**				
	**Accuracy**	**AUC**	**Recall**	**Precision**	**F1-score**	**Accuracy**	**AUC**	**Recall**	**Precision**	**F1-score**
LR	0.86	0.95	0.77	0.90	0.83	0.80	0.86	0.70	0.76	0.73
LR with LASSO	0.78	0.86	0.59	0.85	0.69	0.74	0.80	0.59	0.77	0.67
KNN	0.93	**1.00**	0.84	0.99	0.91	0.80	0.88	0.64	0.89	0.74
SVM linear	0.89	0.92	0.83	0.89	0.86	0.82	0.90	0.75	0.83	0.78
SVM radial	**0.99**	**1.00**	0.98	**1.00**	**0.99**	0.83	0.92	0.76	0.84	0.80
decision tree	0.87	0.92	0.80	0.88	0.84	0.82	0.88	0.75	0.83	0.78
random Forest	0.87	0.86	0.91	0.77	0.84	**0.89**	**0.96**	**0.83**	**0.91**	**0.87**
extreme gradient boosting	0.97	**1.00**	**0.94**	0.99	0.97	0.88	**0.96**	0.81	**0.91**	0.86
neural network	0.76	0.71	0.49	0.89	0.63	0.68	0.70	0.38	0.80	0.52

The model with best performance is given in bold. LR, logistic regression; KNN, K-nearest neighbor; SVM, support vector machine.

## Discussion

The purpose of this study was to assess the predictive performance of different ML algorithms for determining the probability of ACS in T2DM patients. All models had excellent predictive performance, especially the KNN model (AUC, 1.00) and SVM with radial kernel model (AUC, 0.96) for their almost perfect performance in the training dataset and the random forest model (AUC, 0.961) in the testing dataset. The results also suggested that ML algorithms represent promising prospects for identifying ACS.

During the import of data from the medical information recording system, we ensured that all characteristics data were complete. Therefore, data imputation was unnecessary, which may have influenced our results and decreased the accuracy of our models. In addition, original data, without feature selection, were used to establish our models of ML that could retain as many useful characteristics as possible and reduce the loss of significant diagnostic features.

As we all know, T2DM patients have a high incidence of cardiovascular disease. Studies have shown that HbA1c and FBG were found to identify ACS in T2DM patients and improving glucose control decrease the chances of CVD in T2DM patients (23, 24). Besides, prediabetes was highly associated with adverse outcomes in heart failure (25–28). So, it is important to use different ML algorithms to identify the new incidence of ACS in T2DM patients.

Previous studies (29, 30) showed that the TyG index, a new indicator of insulin resistance, was associated with the prognosis of ACS patients or ACS patients after percutaneous coronary intervention. To this end, we investigated whether the TyG index could be used to forecast ACS in T2DM patients. Unfortunately, using logistic regression, although the TyG index was negatively correlated with the probability of new-onset ACS (coefficient = −0.049), it was not statistically significant (*P* = 0.7952). The reason for this remains unclear, and we suspect that TG diluted the impact of TyG index with no statistical significance between T2DM patients and T2DM patients with ACS. Moreover, this index was determined by TG and glucose levels, which were easily influenced by hypotensive or hypolipidemic medications. Therefore, the TyG index may not have prognostic impact in patients with T2DM or T2DM and ACS. The same is true of lipid parameters. We cannot know whether the patient has used hypolipidemic medications or other treatments to reduce blood lipid. So, lipid parameters were also not included in the features of the nomogram.

Using multivariate logistic regression analysis with LASSO, we established a nomogram to predict the probability of new-onset ACS in T2DM patients. The selected features, including a family history of CVD, a history of smoking, a history of drinking, and age were the key factors related to heart disease reported by the American Heart Association in 2021 (4). Many epidemiological (31) and genetic (32–34) evidence suggested that a family history of CVD played a major role in the occurrence of coronary heart disease, and it was also reflected in a position that could not be ignored. Waterpipe drinking and smoking are prevalent among adults. There was sufficient evidence (20, 35) to prove an adverse association between smoking and ACS. According to the report, the 2015 US Dietary Guidelines Advisory Committee summarized that, instead of smoking, proper drinking was considered a healthy diet for cardiometabolic results. Our group had a long history of commitment to the beneficial effects of yellow wine on heart protection (36) and demonstrated that polyphenols and polypeptides in yellow wine inhibited the proliferation and migration of vascular smooth muscle cells (37) to delay the occurrence of cardiovascular events. Thus, a history of drinking was an essential factor in evaluating the probability of ACS in our study. Moreover, AST, as a common biochemical measure, was screened more conveniently and economically than CK-MB, which was a particular measure only if the patients had chest pain. Although AST was not a specific biomarker of ACS, other activities like pulmonary embolism, hepatic failure, and myocarditis would also lead to the increase in AST, while the specificity and sensitivity of AST were less than those of troponin (38, 39) but convenient and fast.

Killip grade accounted for the largest proportion in this nomogram. A high Killip grade often reflects the seriousness of ACS with acute pulmonary edema and cardiogenic shock. A retrospective study also proved that Killip grade was a significant independent predictor in diagnosing ACS (40). Others found that the neutrophil-lymphocyte ratio was an independent predictor of cardiovascular risk because its results were associated with the incidence and mortality of cardiovascular events (41, 42). Although neutrophil-lymphocyte ratio had great potential for predicting CVD, neutrophil count (*P* = 0.0019) performed better than the neutrophil-lymphocyte ratio (*P* = 0.0508) in our study, and the neutrophil-lymphocyte ratio may influence the efficiency of the model. Neutrophil count was chosen instead of neutrophil-lymphocyte ratio, and it performed well in our nomogram model for a single score close to 50. Age (43) played an essential role in the development of CVD, although some studies (44) demonstrated that the incidence of ACS decreased with age in older adults compared with young adults, as organ malfunction and vascular aging would increase. A large-scale clinical trial also confirmed that age was the strongest risk factor for myocardial infarction and stroke in T2DM patients (45).

ML has changed medical services (46). For example, in the event of coronavirus disease 2019 (COVID-2019), ML played a major role in its diagnosis (47), surveillance (48), and mortality risk evaluation (49). The main goal of our study was to evaluate the probability of new-onset ACS using ML. A previous study (50) established a nomogram to predict the probability of ACS, and the AUC values of the training and validation sets were 0.830 and 0.827, respectively. Our study used six other ML algorithms, and our results were better than those of the traditional logistic regression algorithm. The AUC of the training set was 1.00 with the SVM using the radial kernel model, while the AUC of the testing set was 0.96 using the random forest model. The prediction models of ML yielded better discrimination and higher accuracy than the traditional models.

Our research had some advantages. First, we established a nomogram to access the risk of ACS in T2DM patients and the model had high accuracy. Second, we demonstrated the usefulness of ML algorithms in predicting cardiovascular disease. Third, we proved family history of CVD, history of smoking and drinking, aspartate aminotransferase level, age, neutrophil count, and Killip grade were the key features that accelerated the development of ACS in T2DM patients.

### Limitation

The limitations of our study should not be overlooked. First, because of the negligence of the medical staff, body mass index (BMI) data were not available in the medical information recording system. This loss of BMI data may have decreased the model's accuracy. Second, we did not collect information about patients' recent medications, such as hypotensive or hypolipidemic medications, which may cause low blood pressure levels and blood lipid levels. Besides, because the nature of the ML algorithms was “black box,” the clinicians were unable to understand the inherent complexity of the algorithms, which may have led to mistrust. Moreover, our patient sample was not large enough. Thus, we must conduct additional external validation studies and other ML algorithms to update our prediction models.

## Data availability statement

The raw data supporting the conclusions of this article will be made available by the authors, without undue reservation.

## Ethics statement

The studies involving human participants were reviewed and approved by the Ethical Committee of the Shaoxing People's Hospital. The patients/participants provided their written informed consent to participate in this study.

## Author contributions

ZZ: conceptualization, methodology, design of the research, writing, and original draft preparation. ZZ and SS: bioinformatic data collection and analysis. ZZ, JW, JS, and HZ: experimental data collection. ZZ, JS, and SS: experimental data analysis. ZZ and HL: software validation and result interpretation. ZZ and MP: figures preparation. ZZ, JC, and HG: reviewing and revising and editing. All authors approved the final version of the manuscript.

## Funding

This study was supported by the Natural Science Foundation of China (81873120).

## Conflict of interest

The authors declare that the research was conducted in the absence of any commercial or financial relationships that could be construed as a potential conflict of interest.

## Publisher's note

All claims expressed in this article are solely those of the authors and do not necessarily represent those of their affiliated organizations, or those of the publisher, the editors and the reviewers. Any product that may be evaluated in this article, or claim that may be made by its manufacturer, is not guaranteed or endorsed by the publisher.

## References

[1] Ma RCW. Epidemiology of diabetes and diabetic complications in China. Diabetologia. (2018) 61:1249–60. 10.1007/s00125-018-4557-7 29392352

[2] Gregg EW Sattar N Ali MK. The changing face of diabetes complications. Lancet Diabetes Endocrinol. (2016) 4:537–47. 10.1016/S2213-8587(16)30010-9 27156051

[3] Booth GL Kapral MK Fung K Tu JV. Relation between age and cardiovascular disease in men and women with diabetes compared with non-diabetic people: a population-based retrospective cohort study. Lancet. (2006) 368:29–36. 10.1016/S0140-6736(06)68967-8 16815377

[4] Virani SS Alonso A Aparicio HJ Benjamin EJ Bittencourt MS Callaway CW. Heart disease and stroke statistics-2021 Update: a report from the American Heart Association. Circulation. (2021) 143:e254–743. 10.1161/CIR.0000000000000950 33501848 PMC13036842

[5] Sarwar N Gao P Seshasai SR Gobin R Kaptoge S Di Angelantonio E. Diabetes mellitus, fasting blood glucose concentration, and risk of vascular disease: a collaborative meta-analysis of 102 prospective studies. Lancet. (2010) 375:2215–22. 10.1016/S0140-6736(10)60484-9 20609967 PMC2904878

[6] Low Wang CC Hess CN Hiatt WR Goldfine AB. Clinical update: cardiovascular disease in diabetes mellitus: atherosclerotic cardiovascular disease and heart failure in type 2 diabetes mellitus - mechanisms, management, and clinical considerations. Circulation. (2016) 133:2459–502. 10.1161/CIRCULATIONAHA.116.022194 27297342 PMC4910510

[7] Makki N Brennan TM Girotra S. Acute coronary syndrome. J Intensive Care Med. (2015) 30:186–200. 10.1177/0885066613503294 24047692

[8] Roth GA Johnson C Abajobir A Abd-Allah F Abera SF Abyu G. Global, regional, and national burden of cardiovascular diseases for 10 Causes, 1990 to (2015). J Am Coll Cardiol. (2017) 70:1–25. 10.1016/j.jacc.2017.04.052 28527533 PMC5491406

[9] Johnston SS Curkendall S Makenbaeva D Mozaffari E Goetzel R Burton W. The direct and indirect cost burden of acute coronary syndrome. J Occup Environ Med. (2011) 53:2–7. 10.1097/JOM.0b013e31820290f4 21187788

[10] Einarson TR Acs A Ludwig C Panton UH. Prevalence of cardiovascular disease in type 2 diabetes: a systematic literature review of scientific evidence from across the world in 2007–2017. Cardiovasc Diabetol. (2018) 17:83. 10.1186/s12933-018-0728-6 29884191 PMC5994068

[11] Deo RC. Machine learning in medicine. Circulation. (2015) 132:1920–30. 10.1161/CIRCULATIONAHA.115.001593 26572668 PMC5831252

[12] Al-Zaiti S Besomi L Bouzid Z Faramand Z Frisch S Martin-Gill C. Machine learning-based prediction of acute coronary syndrome using only the pre-hospital 12-lead electrocardiogram. Nat Commun. (2020) 11:3966. 10.1038/s41467-020-17804-2 32769990 PMC7414145

[13] Bai Z Lu J Li T Ma Y Liu Z Zhao R. Clinical feature-based machine learning model for 1-year mortality risk prediction of ST-segment elevation myocardial infarction in patients with hyperuricemia: a retrospective study. Comput Math Methods Med. (2021) 2021:7252280. 10.1155/2021/7252280 34285708 PMC8275420

[14] Li YM Jiang LC He JJ Jia KY Peng Y Chen M. Machine learning to predict the 1-year mortality rate after acute anterior myocardial infarction in chinese patients. Ther Clin Risk Manag. (2020) 16:1–6. 10.2147/TCRM.S236498 32021220 PMC6957091

[15] Saini I Singh D Khosla A. QRS detection using K-Nearest Neighbor algorithm (KNN) and evaluation on standard ECG databases. J Adv Res. (2013) 4:331–44. 10.1016/j.jare.2012.05.007 25685438 PMC4293876

[16] Jiang T Gradus JL Rosellini AJ. supervised machine learning: a brief primer. Behav Ther. (2020) 51:675–87. 10.1016/j.beth.2020.05.002 32800297 PMC7431677

[17] Churpek MM Yuen TC Winslow C Meltzer DO Kattan MW Edelson DP. Multicenter comparison of machine learning methods and conventional regression for predicting clinical deterioration on the wards. Crit Care Med. (2016) 44:368–74. 10.1097/CCM.0000000000001571 26771782 PMC4736499

[18] Ambale-Venkatesh B Yang X Wu CO Liu K Hundley WG McClelland R. Cardiovascular event prediction by machine learning: the multi-ethnic study of atherosclerosis. Circ Res. (2017) 121:1092–101. 10.1161/CIRCRESAHA.117.311312 28794054 PMC5640485

[19] Richards BA Lillicrap TP Beaudoin P Bengio Y Bogacz R Christensen A. A deep learning framework for neuroscience. Nat Neurosci. (2019) 22:1761–70. 10.1038/s41593-019-0520-2 31659335 PMC7115933

[20] Lloyd A Steele L Fotheringham J Iqbal J Sultan A Teare MD. Pronounced increase in risk of acute ST-segment elevation myocardial infarction in younger smokers. Heart. (2017) 103:586–91. 10.1136/heartjnl-2016-309595 27899428

[21] Longato E Vettoretti M Di Camillo B. A practical perspective on the concordance index for the evaluation and selection of prognostic time-to-event models. J Biomed Inform. (2020) 108:103496. 10.1016/j.jbi.2020.103496 32652236

[22] Vickers AJ Elkin EB. Decision curve analysis: a novel method for evaluating prediction models. Med Decis Making. (2006) 26:565–74. 10.1177/0272989X06295361 17099194 PMC2577036

[23] Monami M Candido R Pintaudi B Targher G Mannucci E. Improvement of glycemic control in type 2 diabetes: a systematic review and meta-analysis of randomized controlled trials. Nutr Metab Cardiovasc Dis. (2021) 31:2539–46. 10.1016/j.numecd.2021.05.010 34158243

[24] Prattichizzo F de Candia P De Nigris V Nicolucci A Ceriello A. Legacy effect of intensive glucose control on major adverse cardiovascular outcome: Systematic review and meta-analyses of trials according to different scenarios. Metabolism. (2020) 110:154308. 10.1016/j.metabol.2020.154308 32628943

[25] Cai X Liu X Sun L He Y Zheng S Zhang Y. Prediabetes and the risk of heart failure: a meta-analysis. Diabetes Obes Metab. (2021) 23:1746–53. 10.1111/dom.14388 33769672

[26] Cai X Zhang Y Li M Wu JH Mai L Li J. Association between prediabetes and risk of all cause mortality and cardiovascular disease: updated meta-analysis. Bmj. (2020) 370:m2297. 10.1136/bmj.m2297 32669282 PMC7362233

[27] Huang Y Cai X Mai W Li M Hu Y. Association between prediabetes and risk of cardiovascular disease and all cause mortality: systematic review and meta-analysis. Bmj. (2016) 355:i5953. 10.1136/bmj.i5953 27881363 PMC5121106

[28] Mai L Wen W Qiu M Liu X Sun L Zheng H. Association between prediabetes and adverse outcomes in heart failure. Diabetes Obes Metab. (2021) 23:2476–83. 10.1111/dom.14490 34227220

[29] Zhao Q Zhang TY Cheng YJ Ma Y Xu YK Yang JQ. Impacts of triglyceride-glucose index on prognosis of patients with type 2 diabetes mellitus and non-ST-segment elevation acute coronary syndrome: results from an observational cohort study in China. Cardiovasc Diabetol. (2020) 19:108. 10.1186/s12933-020-01086-5 32641127 PMC7341665

[30] Luo E Wang D Yan G Qiao Y Liu B Hou J. High triglyceride-glucose index is associated with poor prognosis in patients with acute ST-elevation myocardial infarction after percutaneous coronary intervention. Cardiovasc Diabetol. (2019) 18:150. 10.1186/s12933-019-0957-3 31722708 PMC6852896

[31] Bachmann JM Willis BL Ayers CR Khera A Berry JD. Association between family history and coronary heart disease death across long-term follow-up in men: the cooper center longitudinal study. Circulation. (2012) 125:3092–8. 10.1161/CIRCULATIONAHA.111.065490 22623718 PMC3631594

[32] Dewey FE Gusarova V Dunbar RL O'Dushlaine C Schurmann C Gottesman O. Genetic and Pharmacologic Inactivation of ANGPTL3 and Cardiovascular Disease. N Engl J Med. (2017) 377:211–21. 10.1056/NEJMoa1612790 28538136 PMC5800308

[33] Helgadottir A Gretarsdottir S Thorleifsson G Hjartarson E Sigurdsson A Magnusdottir A. Variants with large effects on blood lipids and the role of cholesterol and triglycerides in coronary disease. Nat Genet. (2016) 48:634–9. 10.1038/ng.3561 27135400 PMC9136713

[34] Malhotra R Mauer AC Lino Cardenas CL Guo X Yao J Zhang X. HDAC9 is implicated in atherosclerotic aortic calcification and affects vascular smooth muscle cell phenotype. Nat Genet. (2019) 51:1580–7. 10.1038/s41588-019-0514-8 31659325 PMC6858575

[35] Haig C Carrick D Carberry J Mangion K Maznyczka A Wetherall K. Current smoking and prognosis after acute st-segment elevation myocardial infarction: new pathophysiological insights. JACC Cardiovasc Imaging. (2019) 12:993–1003. 10.1016/j.jcmg.2018.05.022 30031700 PMC6547246

[36] Lin H Zhang J Ni T Lin N Meng L Gao F. Yellow Wine Polyphenolic Compounds prevents Doxorubicin-induced cardiotoxicity through activation of the Nrf2 signaling pathway. J Cell Mol Med. (2019) 23:6034–47. 10.1111/jcmm.14466 31225944 PMC6714138

[37] Meng L Liu L Zhou C Pan S Zhai X Jiang C. Polyphenols and polypeptides in chinese rice wine inhibit homocysteine-induced proliferation and migration of vascular smooth muscle cells. J Cardiovasc Pharmacol. (2016) 67:482–90. 10.1097/FJC.0000000000000370 26836482

[38] Johnston CC Bolton EC. Cardiac enzymes. Ann Emerg Med. (1982) 11:27–35. 10.1016/S0196-0644(82)80010-3 7034597

[39] Danese E Montagnana M. An historical approach to the diagnostic biomarkers of acute coronary syndrome. Ann Transl Med. (2016) 4:194. 10.21037/atm.2016.05.19 27294090 PMC4885896

[40] Bugiardini R Yan AT Yan RT Fitchett D Langer A Manfrini O. Factors influencing underutilization of evidence-based therapies in women. Eur Heart J. (2011) 32:1337–44. 10.1093/eurheartj/ehr027 21383003

[41] Shah N Parikh V Patel N Patel N Badheka A Deshmukh A. Neutrophil lymphocyte ratio significantly improves the Framingham risk score in prediction of coronary heart disease mortality: insights from the national health and nutrition examination survey-III. Int J Cardiol. (2014) 171:390–7. 10.1016/j.ijcard.2013.12.019 24388541

[42] Verdoia M Nardin M Gioscia R Negro F Marcolongo M Suryapranata H. Higher neutrophil-to-lymphocyte ratio (NLR) increases the risk of suboptimal platelet inhibition and major cardiovascular ischemic events among ACS patients receiving dual antiplatelet therapy with ticagrelor. Vascul Pharmacol. (2020) 132:106765. 10.1016/j.vph.2020.106765 32681888

[43] Herrington W Lacey B Sherliker P Armitage J Lewington S. Epidemiology of atherosclerosis and the potential to reduce the global burden of atherothrombotic disease. Circ Res. (2016) 118:535–46. 10.1161/CIRCRESAHA.115.307611 26892956

[44] Rosengren A Wallentin L. A KG Behar S Battler A Hasdai D. Sex, age, and clinical presentation of acute coronary syndromes. Eur Heart J. (2004) 25:663–70. 10.1016/j.ehj.2004.02.023 15084371

[45] Bebu I Schade D Braffett B Kosiborod M Lopes-Virella M Soliman EZ. Risk factors for first and subsequent CVD events in type 1 diabetes: The DCCT/EDIC Study. Diabetes Care. (2020) 43:867–74. 10.2337/dc19-2292 32001614 PMC7085803

[46] Schwalbe N Wahl B. Artificial intelligence and the future of global health. Lancet. (2020) 395:1579–86. 10.1016/S0140-6736(20)30226-9 32416782 PMC7255280

[47] Zoabi Y Deri-Rozov S Shomron N. Machine learning-based prediction of COVID-19 diagnosis based on symptoms. NPJ Digit Med. (2021) 4:3. 10.1038/s41746-020-00372-6 33398013 PMC7782717

[48] Li M Zhang Z Cao W Liu Y Du B Chen C. Identifying novel factors associated with COVID-19 transmission and fatality using the machine learning approach. Sci Total Environ. (2021) 764:142810. 10.1016/j.scitotenv.2020.142810 33097268 PMC7550892

[49] Gao Y Cai GY Fang W Li HY Wang SY Chen L. Machine learning based early warning system enables accurate mortality risk prediction for COVID-19. Nat Commun. (2020) 11:5033. 10.1038/s41467-020-18684-2 33024092 PMC7538910

[50] Lyu J Li Z Wei H Liu D Chi X Gong DW. A potent risk model for predicting new-onset acute coronary syndrome in patients with type 2 diabetes mellitus in Northwest China. Acta Diabetol. (2020) 57:705–13. 10.1007/s00592-020-01484-x 32008161 PMC7220880

