# Supplementary material for: Machine learning algorithms identifying the risk of new-onset ACS in patients with type 2 diabetes mellitus: A retrospective cohort study
Source: Front Public Health. 2022 Sep 6;10:947204. doi: 10.3389/fpubh.2022.947204 (PMC9486471; doi:10.3389/fpubh.2022.947204)

Figure S1. Heatmap. The darker the color, the stronger the correlation.

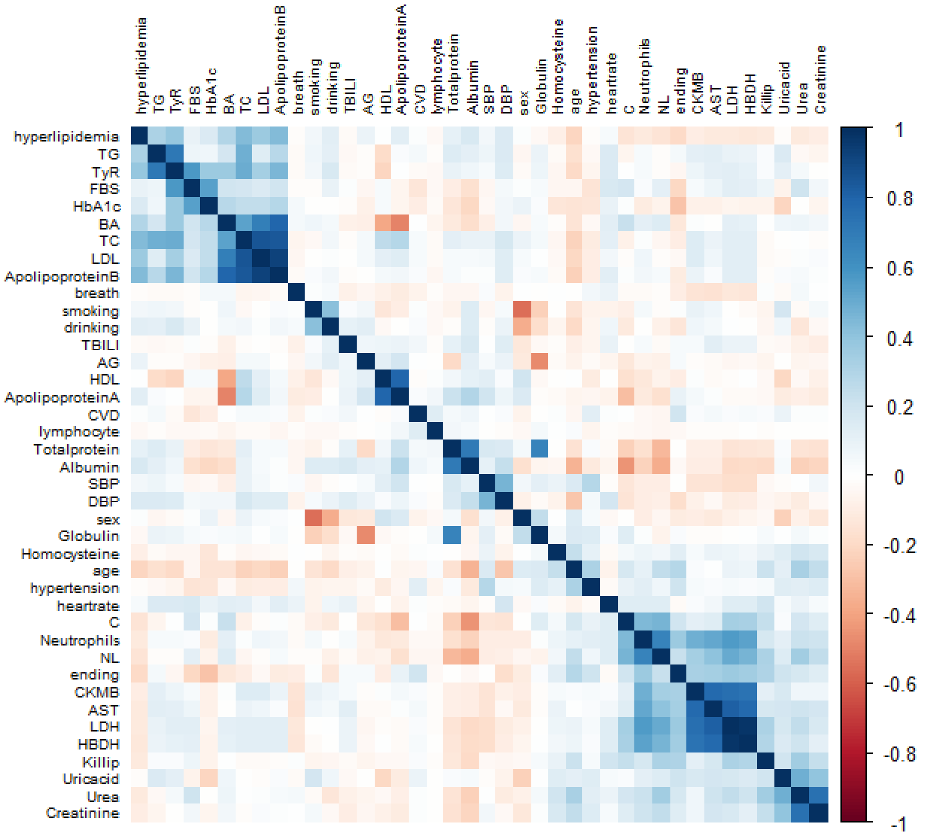

Figure S2. Feature selection by LASSO multivariate logistic regression. (A)The optimal parameter lambda in the LASSO model with 5-fold cross validation by the minimum criteria and 1-SE criteria. (B) LASSO coefficient profiles of all 39 features. Six features with nonzero coefficients by optimal lambda were selected.

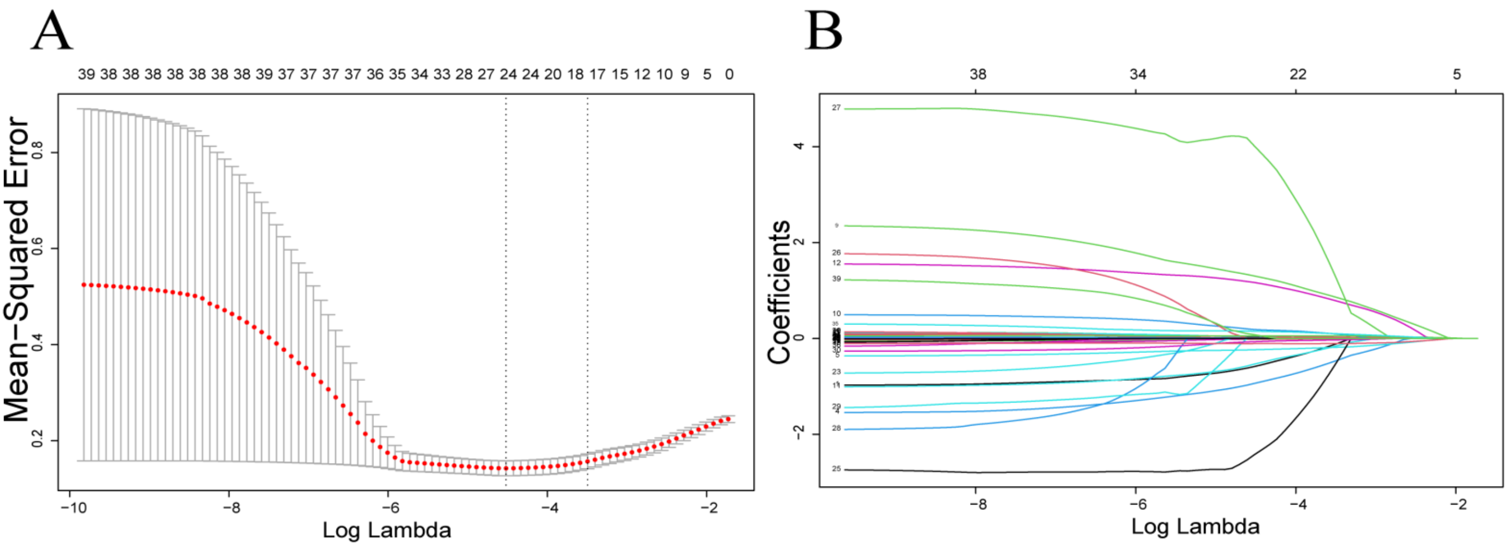

Figure S3. Calibration curves of the nomogram in training dataset (A) and testing dataset (B).

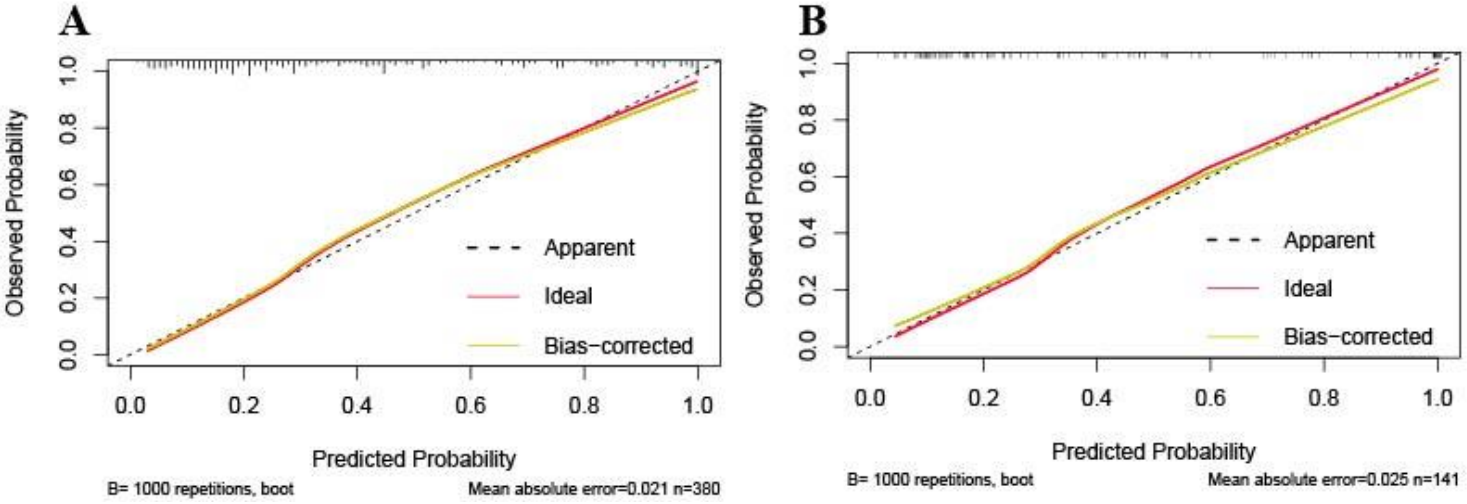

Figure S4. Decision curves for the nomogram in training dataset (A) and testing dataset (B). The red line means the predictive nomogram. The gray line represents all patients are positive and the black line indicates all patients are negative.

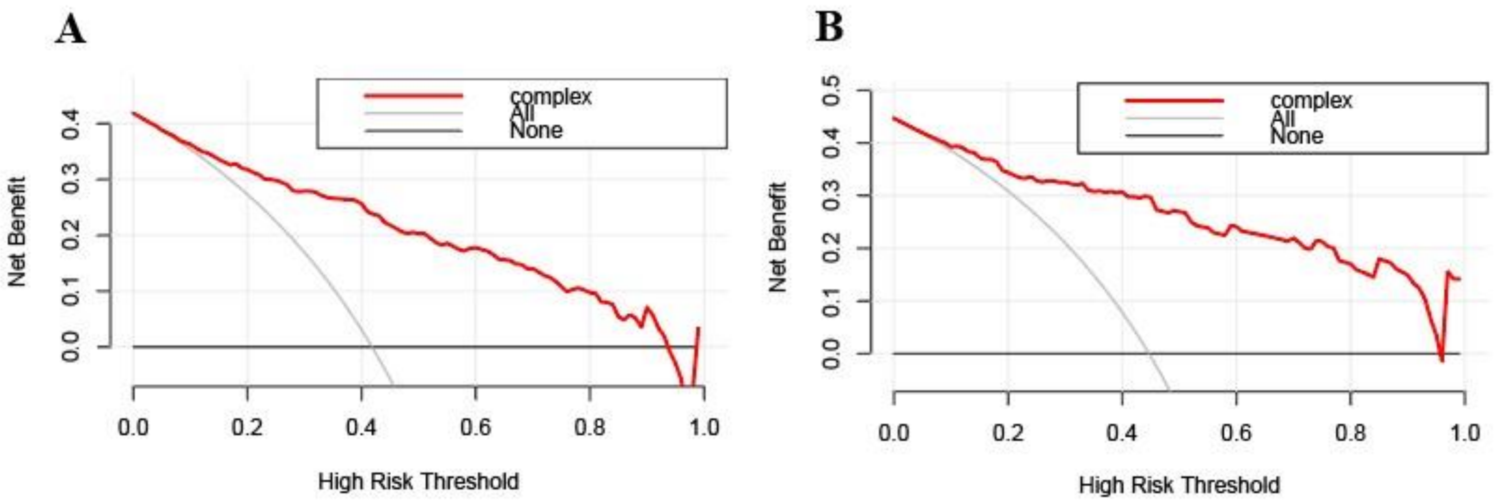

Supplement: Supplementary file 1 [file Data_Sheet_1.pdf]
